# Adherence to the Mediterranean Diet in Saudi Arabia and Its Association with Socioeconomic Status and Depression

**DOI:** 10.3390/medicina60040642

**Published:** 2024-04-17

**Authors:** Majed Alnabulsi, Ahmad Abdullah Imam, Atheer Ahmed Alawlaqi, Fatimah Hussain Alhawaj, Ghazal Fareed Jamjoom, Lina Dakhil Alsaeidi, Fatma El-Sayed Hassan, Shakeel Ahmed Ansari

**Affiliations:** 1 Department of Internal Medicine, General Medicine Practice Program, Batterjee Medical College, Jeddah 21442, Saudi Arabia; majed.alnabulsi@bmc.edu.sa; 2 Internal Medicine Department, College of Medicine, Umm Al-Qura University, Makkah 24382, Saudi Arabia; aaimam@uqu.edu.sa; 3 General Medicine Practice Program, Batterjee Medical College, Jeddah 21442, Saudi Arabia; 110223.atheer@bmc.edu.sa (A.A.A.); 110091.fatimah@bmc.edu.sa (F.H.A.); 120197.ghazal@bmc.edu.sa (G.F.J.); 110310.lina@bmc.edu.sa (L.D.A.); 4 Medical Physiology Department, Kasr Alainy, Faculty of Medicine, Cairo University, Giza 11562, Egypt; fatma.hassan@bmc.edu.sa; 5 General Medicine Practice Program, Department of Physiology, Batterjee Medical College, Jeddah 21442, Saudi Arabia

**Keywords:** mental health, depression, diet, nutrition, Mediterranean

## Abstract

*Background and Objectives:* Several RCTs have reported significant reductions in depression symptoms with the Mediterranean diet (MedDiet), but observational studies have reported inconsistent findings. Moreover, studies have rarely investigated the mediating role of socioeconomic status (SES), including objective material status, in adherence to the MedDiet and its impact on depressive symptoms in the same population. Therefore, this cross-sectional study investigated the relationship between adherence to the MedDiet, socioeconomic factors, and depression severity. *Materials and Methods:* A cross-sectional online survey was conducted between June and December 2022 across Saudia Arabia. The snowball sampling technique was used to recruit participants aged ≥18 years. Mediterranean diet adherence screener (MEDAS) and Patient Health Questionnaire-9 (PHQ-9) were used to assess adherence to the MedDiet and depression severity. An SES index, validated in the Saudi Arabian context, was used to assess SES. The data were analyzed using the Chi-square and Pearson’s correlation tests. *Results:* Only 21% of our study population (*n* = 467) was MedDiet adherent. Adherence was significantly associated with education (*p* = 0.014) but not employment status among traditional SES indicators. Similarly, only television ownership (*p* = 0.009) was associated with MedDiet adherence among the 20 objective material possessions investigated. Nonetheless, the MedDiet-adherent group had a significantly lower PHQ-9 score than the non-adherent group (6.16 ± 0.68 vs. 8.35 ± 0.31, *p* = 0.002). A moderate but significantly negative correlation between MEDAS and PHQ-9 scores (r = −0.16, *p* = 0.001) was noted. *Conclusions:* MedDiet adherence was associated with lower depression severity scores. In addition to education and television ownership, adherence was not associated with any objective indicators of SES.

## 1. Introduction

Since diet and nutrition have an impact on a patient’s health outcomes, they play a crucial role in the pathophysiology, management, and treatment of a variety of biological disorders [1]. Although the exact role played by diet in any given disease is unknown, the evidence that is currently available suggests that food and well-managed nutrition can both prevent and mitigate disease symptoms [2]. Creating the best diet plan to shield patients from disease-related complications during an acute or chronic illness state as well as ensuring adequate and balanced nutrition are two of nutrition’s primary goals [3]. It can be challenging for doctors and patients to stay up-to-date with diet trends, as many of them prioritize weight loss over proper nutrition and overall health. Recommending an appropriate eating style can help patients make positive changes [4]. The Dietary Approaches to Stop Hypertension diet, Weight Watchers, the Mediterranean diet, and the Healthy Eating Plate are dietary patterns that promote health [5]. Obesity, type 2 diabetes mellitus, cancer, and cardiovascular disease can all be prevented with these strategies [6]. Strong evidence that encourages a primary focus on unprocessed foods, fruits and vegetables, plant-based fats and proteins, legumes, whole grains, and nuts supports these dietary patterns. Added sugars should be limited to less than 5% to 10% of daily caloric intake. Vegetables and fruits should make up one-half of each meal. The main sources of carbohydrates should be whole grains, legumes, fruits, and vegetables. Preventing cardiovascular disease, type 2 diabetes, and cognitive decline can be achieved by placing emphasis on monounsaturated fats, like those found in olive oil, avocados, and nuts, and omega-3 fatty acids, like those found in flax, cold-water fish, and nuts [7,8,9,10].

The global rise in chronic diseases, especially cardiometabolic diseases, galvanized scientific investigations into health-promoting food and dietary patterns, among which the Mediterranean diet (MedDiet) is one of the best-studied dietary patterns [11]. The MedDiet is characterized by olive oil as the principal source of dietary fat, vegetables, whole grains, potatoes, beans, nuts, cheese, and fresh fruits as desserts with low-to-moderate fish, poultry, red meat, and wine intake [12]. An umbrella review on the association between the MedDiet and 37 different health outcomes by Dinu et al. [13] reported robust evidence for a lower relative risk of overall mortality, cardiovascular diseases, stroke, cancer, metabolic syndrome and its components (type 2 diabetes, dyslipidemia, and abdominal obesity), neurodegenerative diseases, and depression. The health-promoting effects of the MedDiet are even more significant when combined with physical activity and low tobacco or alcohol consumption [14].

Similarly, the massive increase in mental health burden over the past three decades globally has prompted research on the impact of nutrition and dietary patterns on mental health, especially depression [15]. Several RCTs reported significant reductions in depression symptoms with the MedDiet [16,17]. Similarly, in the GESTAFIT trial, which exclusively recruited pregnant women, MedDiet adherence was associated with lower negative affect, anxiety, and depression, and higher emotional regulation, resilience, and positive affect through the pregnancy [18]. While an RCT is the gold standard for studying the intended effects of exposure, observational studies allow for the complimentary assessment of an outcome in relation to an exposure in the natural setting [19]. Several observational studies have investigated adherence to the MedDiet and the risk of depression, but the findings have been inconsistent. For instance, a meta-analysis of observational studies with the MedDiet as the exposure demonstrated a null association between MedDiet adherence and a risk of depression in pooled data from cohort studies (*n* = 4); in contrast, an inverse significant association was noted among cross-sectional studies (*n* = 9) [20].

Furthermore, both MedDiet adherence [21] and the risk of depression [22] are inversely associated with socioeconomic status (SES). While income, education, and occupation are traditionally used as markers of SES in studies investigating its impact on MedDiet adherence and psychological well-being, a recent study showed better adherence to the MedDiet with every increasing quartile of objective material status (a composite of possession of 16 material items or goods and excluding the traditional SES markers) [21]. Participants in the fourth quartile had a 93% higher probability of MedDiet adherence [21]. Similarly, studies have shown a higher rate of depressive symptoms among those with lower objective material status [23].

However, there is a paucity of studies that have investigated the mediating role of socioeconomic status, including objective material status, in adherence to the MedDiet and its impact on depressive symptoms in the same population. Putting more emphasis on foods rather than on macronutrients can help patients understand what makes a healthy diet. Patients can be helped to follow these recommendations by addressing obstacles to maintaining a healthy diet and making use of the entire health-care team. Therefore, this cross-sectional study investigated the relationship between adherence to the MedDiet, socioeconomic factors, and depression severity in a nationally representative population from the Kingdom of Saudia Arabia (KSA).

## 2. Materials and Methods

### 2.1. Study Design

A cross-sectional online survey was conducted between June and December 2022 across KSA. The survey was open to all Saudi citizens and residents aged 18 years and over. The target sample size of 384 for this cross-sectional survey was calculated using the following considerations: a 95% confidence level, a significance level (α) of 0.05, and a prevalence of 50% was used as a conservative estimate. Choosing a prevalence of 50% provides a sample size that maximizes the required sample size for any given confidence level and margin of error. Based on these considerations, the sample size calculation for a cross-sectional survey typically involves formulas specifically designed for estimating proportions:

*n* = (Z^2^ * *p* * (1 − *p*))/E^2^

where:

- *n* is the required sample size;
- Z is the Z-score corresponding to the desired confidence level (e.g., for a 95% confidence level, Z ≈ 1.96);
- *p* is the estimated prevalence;
- E is the desired margin of error (expressed as a proportion).

Using the given values of Z = 1.96, *p* = 0.5, and a desired margin of error of, for example, 0.05 (5%), the sample size calculation would be as follows:

*n* = (1.96^2^ * 0.5 * (1 − 0.5))/0.05^2^

*n* ≈ 384

However, to account for potential invalid or missing entries, the researchers decided to recruit a larger sample size of 467 participants.

### 2.2. Data Collection

The snowball sampling technique was used to recruit participants as we wanted a nationally representative sample. Medical students circulated survey invitations for the self-administered online questionnaire with medical students in various parts of KSA, who, in turn, shared it on different social media platforms and among co-workers and family members. The link did not permit multiple responses from the same participant to prevent duplicate responses.

The questionnaire comprised four main sections. The first section covered participant characteristics such as age, gender, education, marital status, occupation, area or residence, body weight, height, level of physical activity, smoking status, and chronic illnesses. The second part assessed SES based on an SES index validated by AlOmar et al. [24] for the KSA population. The third section measured adherence to the Mediterranean diet based on the Mediterranean diet adherence screener (MEDAS), which has been adapted for different countries and has proven construct validity [25]. The final part included the Patient Health Questionnaire-9 (PHQ-9) to assess the frequency and severity of depressive symptoms encountered by participants in the past two weeks [26].

Thirty students (excluded from the final sample) were recruited for a pilot study to ensure the clarity and relevance of the questions. No significant changes were made to the questions following the pilot study.

### 2.3. Study Outcomes

The primary study outcome was the adherence rate to the MedDiet in KSA and its association with the severity of depressive symptoms. The secondary outcome of interest was the impact of MedDiet adherence on the prevalence of chronic diseases in this population.

### 2.4. Variables and Data Analysis

The statistical package for social sciences (SPSS) version 18 (SPSS Inc., Chicago, IL, USA) was used for data analysis. Quantitative data were expressed as the arithmetic mean and standard error of the mean. Qualitative data were represented by the relative frequency and percentages (all percentages were calculated from the group). For comparison between groups, the Chi-square test or independent *t*-test were used for qualitative and quantitative data, respectively. Pearson’s correlation coefficient (r) was used to measure the correlation between MEDAS and PHQ-9 scores. A *p*-value of <0.05 was considered statistically significant.

### 2.5. Ethical Considerations

Ethical approval was granted by the Institutional Review Board (IRB) of Batterjee Medical College (reference number RES-2022-0052) before commencing the study. Written consent was prompted as the first question in the survey and was obtained from all participants who wished to complete the survey, which was compatible with our institutional approval.

## 3. Results

Our study population was predominantly female (77.5%) and Saudi nationals (91.9%) (Table 1). Regarding age, most participants were in the 18–25 age group (42.2%), while graduates comprised 53.7% of our study population. Employed (35.8%) and students (35.5) were the most reported employment/occupation status. The western province provided 54% of participants, followed by the eastern (19.7%) and southern (13.5%) provinces. Only 98 of the 467 participants (21%) were MedDiet adherents.

### 3.1. Factors Associated with Adherence to the MedDiet

Table 1 presents the associations between adherence to the MedDiet and the demographic characteristics of the study population. There was no significant association between adherence to the MedDiet and participants’ sex, age, employment, marital status, nationality, province, smoking status, or BMI. However, adherence to the MedDiet was significantly associated with education level (*p* = 0.014) and weekly exercise frequency (*p* = 0.007).

### 3.2. Adherence to the MedDiet and Objective Material Status

Table 2 presents the associations between adherence to the MedDiet and various socioeconomic variables. No significant association was observed between adherence to the MedDiet and home type, housing tenure, car ownership, phone or PC ownership, and availability of the Internet, satellite, video games, video players, and a library. However, adherence to the MedDiet was significantly associated with TV ownership (*p* = 0.009), with 88.8% of adherent subjects owning a TV compared to 76.7% of non-adherent subjects.

### 3.3. Adherence to the Meddiet and Mental Health Status

The PHQ-9 score was significantly lower in the MedDiet-adherent group than in the non-adherent group (6.16 ± 0.68 vs. 8.35 ± 0.31, *p* = 0.002) (Table 3). A more significant proportion of participants in the adherent group had non-minimal depression severity (52% vs. 29.8%), while a lower proportion had mild (24.5% vs. 32.2%), moderate (10.2% vs. 21.7%), and moderately severe (8.2% vs. 10.8%) depression. The prevalence of severe depression was similar in the two groups (5.1% vs. 5.4%). The overall PHQ-9 grading distribution significantly differed between the two groups (*p* < 0.001; Table 3).

**Figure 1:**
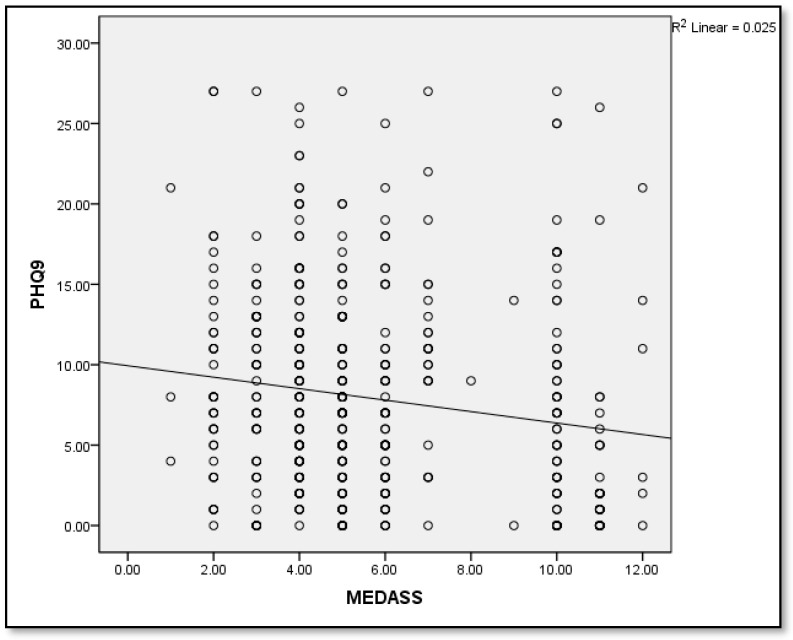
Correlation between MEDAS and PHQ-9.

Pearson’s correlation showed a moderate but significantly negative correlation between MEDAS and PHQ-9 scores (r = −0.16, *p* = 0.001; Figure 1).

### 3.4. Association between Adherence to the Mediterranean Diet and Chronic Diseases

The prevalence of chronic diseases was 19.4% in the MedDiet-adherent group versus 20.9% in the non-adherent group (Table 4). The two groups had no significant differences in the prevalence of specific chronic diseases.

## 4. Discussion

Overall, only a fifth of our study population was MedDiet adherent. Given that the MedDiet has been linked to several health advantages, this emphasizes the necessity for initiatives and measures to support healthy eating habits. Adherence was significantly associated with education but not employment status among traditional SES indicators. Similarly, only television ownership was associated with MedDiet adherence among the 20 objective material possessions investigated. Nonetheless, the population-wide depression severity was significantly lower in the MedDiet-adherent group than in the non-adherent group. The prevalence of non-minimal depression severity was higher, and mild, moderate, and moderately severe depression was lower in the MedDiet-adherent group than in the non-adherent group. Nonetheless, it is important to remember that with 80% of participants being under 46, the current study’s population was comparatively younger. This might account for the higher correlation seen between following the MedDiet and lower depression severity scores compared to earlier research conducted on older individuals. In contrast to previous studies [23], we did not observe any differences in the prevalence of chronic diseases between the MedDiet-adherent group and the non-adherent group, possibly due to the low rate of chronic diseases in the study population (~20% in both groups). This may be due to the young age of the participants and their practice of physical activities, which highlights the significance of lifestyle modification’s positive impact on health.

Several prior observational studies have reported non-significant measures of depression with adherence to the MedDiet, although most of these studies are in geriatric populations [27,28,29]. Different validated scales, such as the Center for Epidemiological Studies-Depression Scale (CES-D) [17], the Geriatric Depression Scale [28], and the Hospital Anxiety and Depression Scale (HADS) [29], were used in these studies to assess depressive symptoms. Similarly, different scales such as the Mediterranean diet scale [17], MedDietScore [28], or author-defined Mediterranean diet [29] were used to measure dietary adherence.

However, in a middle-aged population, Antonogeorgos et al. [30] showed that the positive feeling component of the Zung Self-Rating Depression Scale (ZDRS) was associated with high adherence to MedDietScore and the negative feeling component with low adherence to MedDietScore. Similarly, Rienks et al. [31] showed that adherence to the author-defined Mediterranean diet was associated with 17% lower odds of reporting depressive symptoms using the CES-D scale at the three-year follow-up among middle-aged women. In another study with middle-aged participants, although CES-D scores were similar between low- and high-adherence groups defined by the Mediterranean diet scale, the plant food component of the diet was negatively associated with CES-D scores, trait anxiety, depression, and perceived stress [32].

In contrast to these studies, our study population was younger, with 80% under 46 years old, which could explain the stronger association between the observed lower depression severity scores in the MedDiet-adherent group. Moreover, we used MEDAS and PHQ-9 scores to assess the association between adherence to the MedDiet and depression severity. PHQ-9 is based on the *Diagnostic and Statistical Manual of Mental Disorders* (DSM-IV), which diagnoses about 30% more patients with depressive symptoms than HADS [33]. Studies comparing PHQ-9 and CES-D [34] and PHQ-9 and GDS [35] indicate similar screening performances, although PHQ-9 is shorter and easier to administer than CES-D or GDS. MEDAS is a validated tool for assessing MedDiet adherence with comparable performance to MedDietScore [35].

Furthermore, we observed no significant differences in MedDiet adherence between genders, and the literature is inconsistent on this issue. Higher adherence to the MedDiet has been reported among women in the middle-aged population [30], while higher adherence has been reported among men in a population of university students [36]. Notably, a study that non-discriminately recruited participants aged 18 or older found no significant association between gender and adherence [37]. Moreover, the MedDiet-adherent and non-adherent groups had similar but overweight BMI (25.49 vs. 25.47) classifications. Evidence from prospective studies suggests significant protective effects of MedDiet adherence on long-term overweight/obesity, albeit with a smaller effect size for overweight risk [38]. However, we noted higher MedDiet adherence among individuals who regularly exercise, which is consistent with previous studies [39].

## 5. Conclusions and Prospectives

In conclusion, the research population in Saudi Arabia had a poor adherence rate to the MedDiet, which emphasizes the importance of programs that support healthy eating habits. This study offers proof of a connection between following the MedDiet and a decreased incidence of severe depression, indicating a possible function of the MedDiet in enhancing mental health. To gain a deeper understanding of the therapeutic effects of MedDiet adherence on mental health outcomes and the prevention of chronic diseases in a variety of groups, more research is required, including longitudinal and interventional studies.

## 6. Limitations of This Study

Still, there are some limitations to our study which limit the data’s generalizability. (a) In terms of study design, to further explore causal relationships, longitudinal or interventional studies, rather than cross-sectional studies, are needed to investigate the causal effects of diet on socioeconomic status and depression. (b) There is a lack of exploration of age/gender-specific associations with our outcomes; therefore, more studies with a more evenly distributed sample across age/gender groups would provide a more comprehensive understanding of the relationship between age/gender-related characteristics and Mediterranean diet adherence in Saudi Arabia and we suggest using focused recruiting or oversampling methods for future research. (c) Socioeconomic status, physical activity, and educational levels might potentially confound the outcomes of our study; thus, future research, including longitudinal studies and interventions, to further explore the complex relationships between these factors, dietary patterns, and mental health outcomes is warranted. (d) This study implemented a short-term assessment of depression; a long-term depression assessment would be more reliable. (e) There exists a probability of changes in quantities or qualities of different components/frequencies of the MedDiet. (f) There was a huge difference between the MedDiet-adherent and non-adherent participants’ numbers. (g) The data were in the form of subjective responses to the questionnaires, which carries the possibility of measurement bias. (h) The participant recruitment process was limited to those with access to the Internet, although multiple recruitment strategies could improve the diversity of the sample. However, we believe that this only has a marginal impact on our data as about 93% of people in KSA had access to the Internet, and 97.9% owned mobile phones in 2021 [40]. In addition, there is an inherent risk of unknown confounding variables and sampling bias in any observational study [9]. Addressing such limitations in future research would improve the validity and applicability of our findings.

## Figures and Tables

**Table 1:** Association between adherence to Mediterranean diet and patient characteristics.

Variables	Overall *n* (%)	Adherent *n* (%)	Non-Adherent *n* (%)	Test	*p*
Overall	467 (100)	98 (21)	369 (79)		
Sex					
Male	105 (22.5)	19 (19.4)	86 (23.3)	0.68	0.41
Female	362 (77.5)	79 (80.6)	283 (76.7)		
Age group					
18–25	197 (42.2)	40 (40.8)	157 (42.5)	5.57	0.14
26–35	93 (19.9)	24 (24.5)	69 (18.7)		
36–45	85 (18.2)	11 (11.2)	74 (20.1)		
≥46	92 (19.7)	23 (23.5)	69 (18.7)		
Education					
None	4 (0.9)	3 (3.1)	1 (0.3)	12.57	0.014 *
High school	131 (28.1)	23 (23.5)	108 (29.3)		
Graduate	251 (53.7)	52 (53.1)	199 (53.9)		
Diploma	39 (8.4)	6 (6.1)	33 (8.9)		
Postgraduate	42 (9)	14 (14.3)	28 (7.6)		
Employment/occupation status					
Unemployed	3 (0.6)	1 (1)	2 (0.5)	6.51	0.58
Employed	167 (35.8)	39 (39.8)	128 (34.7)		
Student	166 (35.5)	36 (36.7)	130 (35.2)		
Intern	2 (0.4)	0 (0)	2 (0.5)		
Housewife	84 (18)	11 (11.2)	73 (19.8)		
Retired	35 (7.5)	9 (9.2)	26 (7)		
Searching for work	7 (1.5)	1 (1)	6 (1.6)		
Graduated	1 (0.2)	0 (0)	1 (0.3)		
Private Work	2 (0.4)	1 (1)	1 (0.3)		
Marital status					
Single	209 (44.8)	42 (42.9)	167 (45.3)	1.04	0.79
Married	237 (50.7)	52 (53.1)	185 (50.1)		
Widowed	3 (0.6)	0 (0)	3 (0.8)		
Divorced	18 (3.9)	4 (4.1)	14 (3.8)		
Nationality					
Saudi	429 (91.9)	89 (90.8)	340 (92.1)	0.18	0.67
Non-Saudi	38 (8.1)	9 (9.2)	29 (7.9)		
Province					
Southern	63 (13.5)	21 (21.4)	42 (11.4)	8.46	0.08
Northern	35 (7.5)	4 (4.1)	31 (8.4)		
Central	25 (5.4)	4 (4.1)	21 (5.7)		
Western	252 (54)	52 (53.1)	200 (54.2)		
Eastern	92 (19.7)	17 (17.3)	75 (20.3)		
Smoking	102 (21.8)	24 (24.5)	78 (21.1)	0.51	0.47
Exercise					
Do not exercise	234 (50.1)	34 (34.7)	200 (54.2)	12.08	0.007 *
Once per week	86 (18.4)	22 (22.4)	64 (17.3)		
Twice per week	66 (14.1)	19 (19.4)	47 (12.7)		
≥3 times per week	81 (17.3)	23 (23.5)	58 (15.7)		
BMI (kg/m^2^), mean ± SD	25.56 ± 0.27	25.49 ± 0.53	25.57 ± 0.32	0.12	0.90

*: *p* < 0.05 means statistically significant.

**Table 2:** Association between adherence to Mediterranean diet and objective material status.

Variables	Overall *n* (%)	Adherent *n* (%)	Non-Adherent *n* (%)	Test	*p*
Home type					
Villa	152 (32.5)	30 (30.6)	122 (33.1)	9.10	0.11
Apartment	168 (36)	30 (30.6)	138 (37.4)		
Traditional House	105 (22.5)	30 (30.6)	75 (20.3)		
Floor in Traditional House	40 (8.6)	7 (7.1)	33 (8.9)		
Duplex	1 (0.2)	1 (1)	0 (0)		
Rented Apartment	1 (0.2)	0 (0)	1 (0.3)		
Tenure of housing					
Owned	342 (73.2)	73 (74.5)	269 (72.9)	3.60	0.16
Rented	112 (24)	25 (25.5)	87 (23.6)		
Provided	13 (2.8)	0 (0)	13 (3.5)		
Car ownership					
No car	249 (53.3)	51 (52)	198 (53.7)	0.35	0.83
One car	155 (33.2)	32 (32.7)	123 (33.3)		
Two or more cars	63 (13.5)	15 (15.3)	48 (13)		
Phone ownership	447 (95.7)	93 (94.9)	354 (95.9)	0.20	0.65
TV ownership	370 (79.2)	87 (88.8)	283 (76.7)	6.86	0.009 *
PC ownership	356 (76.2)	81 (82.7)	275 (74.5)	2.82	0.09
Internet	377 (80.7)	74 (75.5)	303 (82.1)	2.17	0.14
Satellite	84 (18)	19 (19.4)	65 (17.6)	0.16	0.68
Video games	137 (29.3)	26 (26.5)	111 (30.1)	0.47	0.49
Video player	83 (17.8)	15 (15.3)	68 (18.4)	0.51	0.47
Library	124 (26.6)	25 (25.5)	99 (26.9)	0.08	0.78

*: *p* < 0.05 means statistically significant.

**Table 3:** Association between adherence to Mediterranean diet and mental health status.

	Total	Adhered	Did Not Adhere	Test	*p*
	Mean ± SD	Mean ± SD	Mean ± SD		
MEDAS	5.74 ± 0.13	10.45 ± 0.06	4.49 ± 0.07	40.43	<0.001 *
PHQ-9	7.89 ± 0.29	6.16 ± 0.68	8.35 ± 0.31	3.12	0.002 *
PHQ-9 Grading	n (%)	n (%)	n (%)	Test	*p*
Non-Minimal (0–4)	161 (34.5)	51 (52)	110 (29.8)	18.46	<0.001 *
Mild (5–9)	143 (30.6)	24 (24.5)	119 (32.2)		
Moderate (10–14)	90 (19.3)	10 (10.2)	80 (21.7)		
Moderately Severe (15–19)	48 (10.3)	8 (8.2)	40 (10.8)		
Severe (20–27)	25 (5.4)	5 (5.1)	20 (5.4)		

*: *p* < 0.05 means statistically significant.

**Table 4:** Association between adherence to Mediterranean diet and patients’ chronic diseases.

Variables	Overall *n* (%)	Adherent *n* (%)	Non-Adherent *n* (%)	Test	*p*
Presence of Chronic Disease					
No	371 (79.4)	79 (80.6)	292 (79.1)	7.20	0.10
Yes	96 (20.6)	19 (19.4)	77 (20.9)		
Type of chronic disease					
Diabetes Mellitus (DM)	19 (4.1)	2 (2)	17 (4.6)	14.13	0.74
Gestational Diabetes	1 (0.2)	1 (1)	0 (0)		
Hypertension (HTN)	17 (3.6)	5 (5.1)	12 (3.3)		
DM, HTN, and Hyperlipidemia	13 (2.8)	4 (4.1)	9 (2.4)		
Chronic Heart Disease	3 (0.6)	1 (1)	2 (0.5)		
Autoimmune Disease	9 (1.9)	1 (1)	8 (2.2)		
G6PD Deficiency	2 (0.4)	1 (1)	1 (0.3)		
Irritable Bowel Syndrome and Polycystic Ovary Syndrome	2 (0.4)	0 (0)	2 (0.5)		
Rheumatoid Arthritis, Knee Affection, Psoriasis, Anemia, and Depression	1 (0.2)	0 (0)	1 (0.3)		
Asthma and Sinusitis	3 (0.6)	0 (0)	3 (0.8)		
Thyroid Disease	4 (0.9)	0 (0)	4 (1.1)		

## Data Availability

Data is contained within the article.
